# Dietary diversity, frailty, and cognitive function in community-dwelling older adults: a cross-sectional study from Türkiye

**DOI:** 10.3389/fmed.2026.1787808

**Published:** 2026-04-29

**Authors:** Şebnem Özgen Özkaya, Volkan Özkaya

**Affiliations:** Kutahya Health Sciences University, Kutahya, Türkiye

**Keywords:** activities of daily living, cognitive decline, dietary diversity, frailty, older adults, phytonutrient

## Abstract

**Background:**

With the global increase in the older adults population, early detection of health risks and the promotion of healthy aging have become increasingly important. Accordingly, the aim of this study is to investigate the relationship between dietary diversity and frailty, cognitive function, activities of daily living, phytonutrients intake and nutritional status in Turkish older adults.

**Methods:**

This cross-sectional study included a total of 1744 community-dwelling individuals aged 65 and older from different geographical regions of Türkiye. Sociodemographic characteristics and certain anthropometric measurements of the participants were recorded. The Dietary Diversity Score (DDS), Mini Nutritional Assessment Short Form (MNA-SF), two-day 24-h dietary recall for dietary intake, Phytonutrient Index, Katz Index of Independence in Activities of Daily Living (Katz ADL), Edmonton Frailty Scale (EFS), and Standardized Mini Mental State Examination (SMMSE) were used.

**Results:**

Of the participants, 3.3% were classified as severely frail, 20.7% were at risk of malnutrition, 21.7% had moderate to severe cognitive impairment, and 18.6% had low DDS. A significant positive correlation was found between DDS and energy intake (r = 0.453, *p* < 0.01), carbohydrate (g) (*r* = 0.341, *p* < 0.01), protein (g) (*r* = 0.434, *p* < 0.01), fat (g) (*r* = 0.438, *p* < 0.01), phytonutrient intake (*r* = 0.296, *p* < 0.01), nutritional status (MNA-SF: *r* = 0.088, *p* < 0.01), and cognitive function (SMMSE: *r* = 0.075, *p* < 0.01). A negative correlation was found between DDS and frailty (*r* = −0.060, *p* < 0.05). However, according to the multinomial logistic regression model, the associations between DDS and MNA-SF, SMMSE, ADL, and frailty did not persist. Additionally, participants in the low DDS group were significantly less likely to exhibit high phytonutrient intake (*p* < 0.001).

**Conclusion:**

No significant association was found between dietary diversity and nutritional status, activities of daily living, functional and cognitive frailty in the older adults Turkish population. It is considered that regular monitoring of nutritional status and interventions aimed at increasing dietary diversity in the older adults population may promote healthy aging.

## 1 Introduction

One of the most significant demographic shifts observed today in both developed and developing countries is the increasing proportion of the older adults population. In 2022, approximately 10% of the global population was aged 65 and over and this ratio is expected to rise to 12% by 2030 and to 16% by 2050 (1, 2). Population aging, recognized as a global public health concern, also affects Türkiye. According to the Turkish Statistical Institute, the proportion of individuals aged 65 and over, which was 7.5% in 2012, is expected to reach 12.9% by 2030 and 22.6% by 2060 (1, 3). In recent years, researchers have increasingly focused on promoting successful aging among older adults. Successful aging is defined as the absence of disease and disability, the presence of high cognitive, mental, and physical functioning, active engagement in life activities, and a positive psychological adaptation to advanced age (4, 5). According to the World Report on Ageing and Health, most health problems observed in old age stem from chronic diseases, which can be prevented or delayed through healthy lifestyle behaviors such as balanced nutrition and physical activity (6). Frailty is a geriatric syndrome characterized by a decreased capacity to maintain homeostasis due to loss of biological reserves and disruption of physiological mechanisms, and it becomes more common with advancing age. It is also associated with an increased risk of adverse health outcomes such as falls, hospitalization, dependence, and mortality. Due to global aging, frailty is considered a significant public health problem (7, 8). Although the frequency of frailty varies depending on the assessment method used and the country in which the study is conducted, a meta-analysis involving 62 countries reported a physical frailty rate of 12% and a pre-frailty rate of 46% (8). According to the FrailTURK Project, conducted with older adults people in 13 centers in Türkiye, 39.2% of participants were found to be frail and 43.3% were pre-frail (7).

Another common issue associated with the rapid aging of the population is the decline in cognitive functions. Cognitive decline threatens the wellbeing of older adults and their families due to the current lack of significantly effective pharmacological treatments, making the prevention of cognitive impairment in older adults increasingly important (9). Focusing on modifiable risk factors is critically important for the prevention of cognitive decline. Nutrition plays a key role in both the prevention of frailty and the protection against cognitive impairment and decline (9, 10). Inadequate nutrition, multiple nutrient deficiencies, monotonous diets, and low dietary diversity are known to play a role in the pathogenesis of sarcopenia, a core component of frailty. Dietary diversity is considered an important component and practical indicator of diet quality (11). Studies have reported that increasing the consumption of fruits and vegetables may help prevent frailty (10, 12). Older adults who maintain a high level of dietary diversity have been reported to have lower frailty, and each point increase in dietary diversity reduces the risk of frailty by 5% (11, 13). Among women aged 60 and over who were followed for more than 22 years, a one standard deviation increase in diet quality score was associated with a 10% reduction in frailty risk (14). In a study conducted in Taiwan, higher dietary diversity among older adults was associated with a lower frequency of cognitive impairment, while those with both frailty and low dietary diversity were found to have a significantly higher risk of cognitive decline (15). Similar findings were reported in A Community-Based, Nationwide Cohort Study conducted in China, where higher dietary diversity was shown to have beneficial effects on cognitive functions and their subdomains, even in the final stages of life, particularly among women and illiterate individuals over the age of 80 (9).

Phytonutrients are defined as bioactive compounds found in and/or derived from plants that provide health benefits. An increase in dietary diversity can contribute to a higher consumption of fruits, vegetables, whole grains, nuts, and other plant-derived foods rich in phytonutrients and other bioactive compounds. Diets rich in phytonutrients have the potential to influence multiple aging-related mechanisms, including inflammation, metabolism, and cellular repair (16). In this regard, it is reported that phytonutrient-rich diets improve physical performance, cognitive function, attention, neuroprotection, language, and memory in older individuals and may also reduce levels of anxiety and depression (16, 17). Despite these potential effects, studies on the role of phytonutrients intake on frailty and cognitive function in the older adults are limited.

Although evidence regarding the role of dietary diversity in healthy aging is increasing, findings remain heterogeneous and mostly focus on a single health outcome. Comprehensive studies that simultaneously address the relationships between dietary diversity, frailty, cognitive function, activities of daily living, phytonutrients intake, and nutritional status are still limited. It is known that dietary diversity, considered an important component of healthy aging, can affect nutritional status and thus be potentially protective against frailty and cognitive functions. Therefore, evaluating these variables together can provide a more comprehensive and integrated understanding of the role of dietary diversity in the multidimensional aspects of health in older adults. For this reason, this study aims to evaluate the relationship between dietary diversity and frailty, cognitive function, activities of daily living, phytonutrients intake, and nutritional status in Turkish older adults.

## 2 Materials and methods

### 2.1 Study design and participants

In this study, a cross-sectional design was employed. Data were collected through face-to-face interviews with community-dwelling individuals aged 65 and over between January and April 2025. Participants were selected from 16 different provinces in the Central Anatolia, Marmara, and Aegean regions of Türkiye. To ensure geographical representation, the sample was distributed relatively equally across these three regions. The minimum sample size for this study was determined *a priori* using G*Power software, based on global reference effect sizes regarding the association between the DDS and functional outcomes. According to a major population-based cohort study by Zhang et al. (18), the hazard ratio (which approximates the odds ratio for relatively rare outcomes) for developing ADL was reported as 0.50 for the highest DDS tertile compared to the lowest tertile. To detect a similar expected effect size (OR = 0.50) with an 85% statistical power and a two-sided significance level of α = 0.05, the minimum required sample size was calculated to be approximately 600 individuals, accounting for the baseline prevalence and the unequal distribution among DDS groups. This final achieved sample size provides greater than 95% statistical power to detect the aforementioned literature-based effect sizes, thereby effectively minimizing the probability of a Type II error for the primary outcomes. Participants were selected via convenience sampling from volunteers, ensuring a balanced distribution of male and female. Potential participants were invited to take part in the study through face-to-face contact in various public settings (e.g., mosques, coffeehouses, parks, outdoor cafes, and local bazaars) by trained researchers. Following standardized verbal and written explanations of the study protocols—including its purpose, procedures, confidentiality, and the voluntary nature of participation—individuals who provided written informed consent and met the eligibility criteria were enrolled in the study.

**FIGURE 1:**
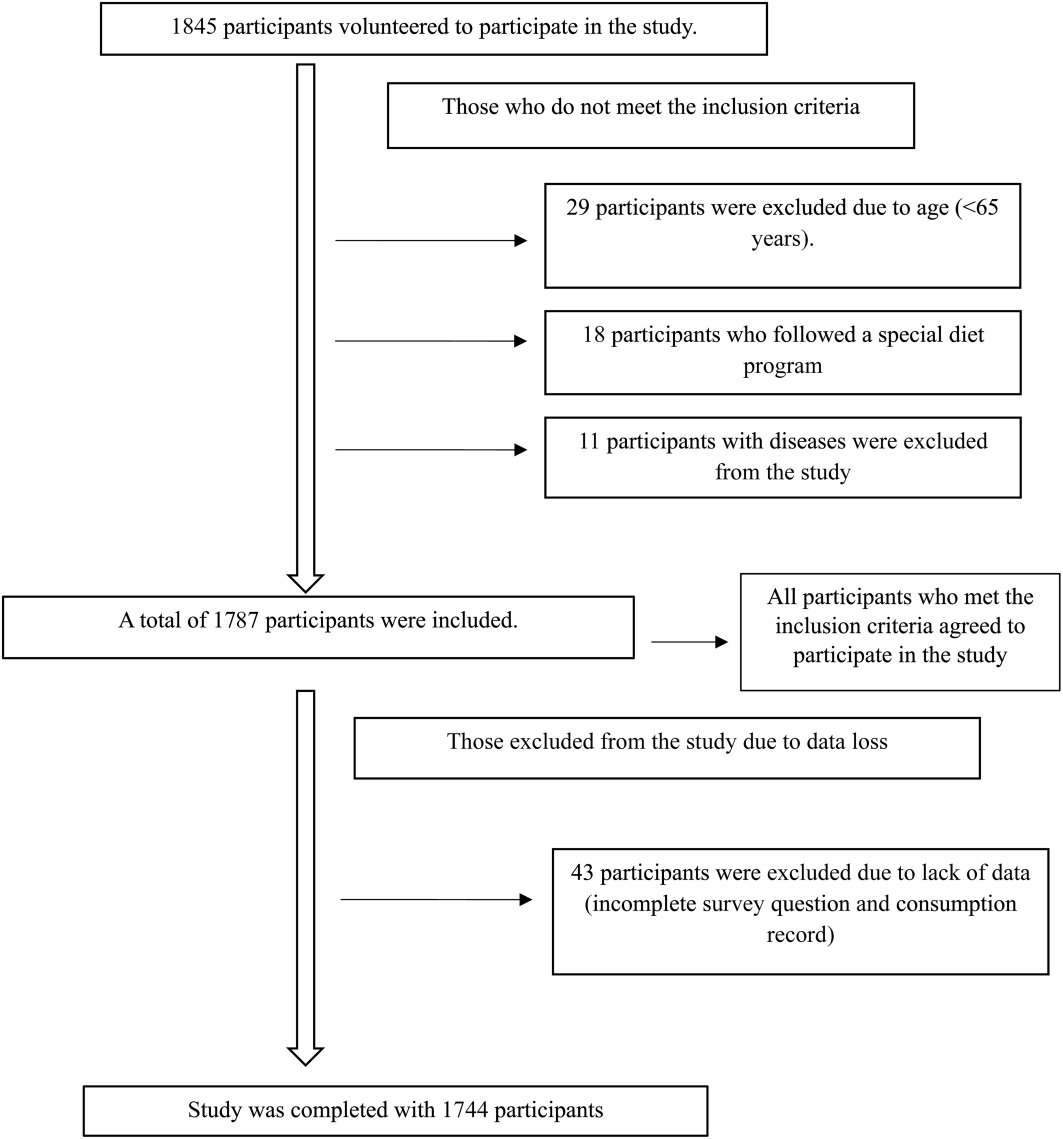
Participant recruitment flowchart.

Inclusion criteria were being community-dwelling, aged 65 or older, and willing to participate voluntarily. Exclusion criteria included residing in institutions such as nursing homes or elder care facilities; having neurological disorders such as dementia or Alzheimer’s disease; having a condition that restricts food intake, such as cancer or malnutrition; following a special diet program (e.g., weight loss diet, diabetic diet); being bedridden; or having a physical disability (Figure 1). Ethical approval for the study was obtained from the Non-Interventional Clinical Research Ethics Committee of Kutahya Health Sciences University (approval number: 2024/13–36, dated November 18, 2024). The study was conducted in accordance with the provisions of the Helsinki Declaration, and informed consent was obtained from all participants.

The first section of the questionnaire included descriptive information, sociodemographic and anthropometric measurements, medical history, and questions regarding sleep and dietary habits. The second section included questions about 2-day 24-h dietary recall, nutritional status, frailty, cognitive function status, dietary diversity, activities of daily living and phytonutrients intake.

### 2.2 Anthropometric measurements

Height (cm) was measured in the Frankfort plane to the nearest 0.1 cm using a Mesilife-13539 portable stadiometer. Body weight (kg) was measured without shoes to the nearest 0.1 kg using a professional scale. Participants’ body mass index (BMI) was calculated using the standard formula: body weight (kg) divided by height squared (m^2^). Waist circumference was measured in the fasting state with an empty bladder, feet together, standing upright, and arms crossed over the chest. Measurements were obtained using an inelastic tape measure at the midpoint between the lower border of the costal arch and the crista iliaca. Hip circumference was measured with the participant standing upright, at the point of maximum gluteal muscle prominence, using a non-stretching measuring tape. Mid-upper arm circumference (MUAC) was measured using a non-stretch measuring tape between the lateral projection of the acromion process of the scapula and the inferior margin of the olecranon process of the ulna, with the participant in a standing position. A waist circumference of ≥ 94 cm in men and ≥ 80 cm in women indicates an increased risk for chronic diseases (19, 20). Waist-to-hip ratio and BMI classifications were evaluated according to World Health Organization (WHO) criteria (20).

### 2.3 Assessment of nutritional status

The Mini Nutritional Assessment Short-Form (MNA-SF) was used to evaluate participants’ nutritional status (21). The MNA-SF is an instrument for screening malnutrition in older adults. The validity and reliability assessment of the Turkish version of the MNA-SF was conducted by Sarıkaya et al. (22). The scale includes questions on changes in food intake (such as appetite loss, digestive issues, or chewing and swallowing difficulties), recent weight loss, mobility, psychological stress or acute illness, neuropsychological problems, and body mass index. Each item is scored from 0 to 3, yielding a maximum total score of 14. The MNA-SF score was categorized into three groups: well-nourished (MNA-SF > 11), at-risk (MNA-SF 8–11), and malnourished (MNA-SF < 8).

### 2.4 Assessment of dietary intake

Participants’ dietary intake was assessed by trained researchers using a 24-h dietary recall form covering two consecutive days, 1 weekday and 14 weekend day. To enhance the accuracy of the dietary records, participants were given detailed instructions on portion sizes in advance using the Food and Meal Photographic Catalogue (23). Dietary records were analyzed using the database of the Nutrition Information System (BeBiS) software, and participants’ energy and nutrient intakes were evaluated in accordance with the Turkey Dietary Guideline (TUBER-2022) (24).

### 2.5 Assessment of dietary diversity

The Dietary Diversity Score (DDS) is an index used to reflect diet quality (25). 2-day dietary records were used to calculate the dietary diversity score of the participants. Dietary diversity was based on the evaluation of five food groups namely grains, vegetables, fruits, meat, and dairy. Within these five groups, 23 subcategories of foods were scored. Fats and sugars were excluded from the DDS calculation. Each main food group consists of a different number of subgroups -for example, the grain group has seven subgroups. The maximum score for any main group on the scale is 2, meaning that exhausting all sub-groups yields two points. The highest total score that can be obtained on the scale is 10. According to the threshold values used in the DDS classification, scores < 3.5 are classified as low diversity, scores between 3.5 and 6.5 as medium diversity, and scores above 6.5 as high diversity (26).

### 2.6 Assessment of phytonutrient intake

Phytonutrient intake was assessed using a phytonutrient index (PI). According to the PI, a diet rich in fruits, vegetables, legumes, whole grains, seeds, nuts, olive oil, soy sources, and wine is considered high in phytonutrients. The Phytonutrient Index (PI) is determined by calculating the ratio of caloric intake from phytochemical-rich foods to total daily energy intake, expressed as a percentage (PI = [phytochemical calories/total calories] × 100). The PI scores were determined by analyzing participants’ 2-day dietary intake records. Total scores were categorized as follows: 0–19, negligible; 20–39, low; 40–59, moderate; and ≥ 60, high phytonutrients intake (27).

### 2.7 Assessment of activities of daily living

Activities of daily living were assessed using the Katz Index of Independence in Activities of Daily Living (Katz ADL), developed by Katz et al. (28). The Turkish version of the index was validated by Pehlivanoğlu et al. (28). Participants selected one of two response options -dependent or independent- for each function (bathing, dressing, toileting, transferring, continence, feeding) on the scale. The maximum score obtainable from the Katz ADL is 6. A score of 6 was interpreted as full functionality (independent), scores between 3 and 5 as moderate functional impairment (partially dependent), and scores of ≤ 2 as severe functional impairment (dependent) (29).

### 2.8 Assessment of frailty

Frailty was assessed using the Edmonton Frailty Scale (EFS), developed by Rolfson et al. (30). The validity and reliability of the Turkish version of the scale were established by Aygör et al. (31). The scale comprises 11 items covering 9 parameters, namely: general health status, cognitive function, social support, functional independence, medication use, malnutrition, psychological status, incontinence, and functional performance. The scores obtainable from the scale range from 0 to 17. A score of 0–4 was considered as not frail, 5–6 as apparently vulnerable, 7–8 as mildly frail, 9–10 as moderate frail, and scores of 11 or higher as severely frail (31).

### 2.9 Assessment of cognitive function

The validity and reliability of the Standardized Mini Mental State Examination (SMMSE)’s Turkish version were established by Güngen et al. (32). The scale composed of 11 items measuring five areas of cognitive function: orientation, registration, attention and calculation, recall, and language. The maximum score obtainable from the test is 30. A score of 23 or below is considered indicative of cognitive impairment. Based on the scores, 0–9 indicates severe cognitive impairment, 10–19 moderate impairment, 20–23 mild impairment, and 24–30 is interpreted as no cognitive impairment (32).

### 2.10 Statistical analysis of the data

In the study, the mean and standard deviation (Mean ± SD) were provided for continuous variables that followed a normal distribution, while the median, minimum, and maximum values were provided for variables that did not follow a normal distribution. Categorical variables were presented as frequency (n) and percentage (%). For comparisons between more than two independent groups, the Kruskal-Wallis H test was used for variables that did not follow a normal distribution. When a significant difference was detected, Dunn’s *post hoc* test with Bonferroni correction was applied for pairwise comparisons to account for multiple testing and control the Type I error rate. The Pearson Chi-square test was used for comparisons between categorical variables; where the expected cell counts were small, the Fisher-Freeman-Halton exact test was used for tables larger than 2 × 2. Additionally, predictors of functional independence, frailty status, and nutritional quality were identified using Multinomial Logistic Regression analysis. In these models, DDS groups were treated as the primary independent variable to determine their association with health outcomes, while adjusting for significant covariates such as education, income, chronic disease and tooth loss intake; with results reported as odds ratios (OR) and 95\% confidence intervals (CI). All dietary intake were energy-adjusted (1,000 kcal) prior to statistical analyses. Statistical significance was set at *p* < 0.05, and statistical analysis was performed using SPSS version 22.0 (SPSS Inc., Chicago, IL, United States).

**TABLE 1:** Basic characteristics of the participants according to dietary diversity score.

	Dietary Diversity Score				
Variables	Low *n*:325,18.6%	Moderate *n*:1268, 72.7%	High *n*:151, 8.6%	Total *n*:1744	p
Gender					
Female	175(53.8)	626(49.2)	75(49.6)	876(50.2)	0.351^a^
Male	150(46.2)	642(50.8)	76(50.4)	868(49.8)	
Age					
65–69	177(54.5)	622(49.1)	77(51.0)	876(50.2)	
70–74	79(24.3)	350(27.6)	45(29.8)	474(27.2)	0.511^a^
75–79	44(13.6)	192(15.1)	16(10.6)	252(14.4)	
80 or above	25(7.6)	104(8.2)	13(8.6)	142(8.1)	
Education					
Literate (no formal education)	65(20.1)	254(20.0)	25(16.6)	344 (19.7)	< 0.001^a^
Primary or middle school	228(70.1)	752(59.3)	84(55.6)	1064(61.0)	
High school	29(8.9)	182(14.4)	31(20.5)	242(13.9)	
University and above	3(0.9)	80(6.3)	11(7.3)	94(5.4)	
Household Income					
Below minimum wage	136(41.8)	457(36.1)	46(30.5)	639(36.7)	
Minimum wage	107(33.0)	388(30.6)	39(25.8)	534(30.6)	
Twice the minimum wage	70(21.5)	321(25.3)	45(29.8)	436(25.0)	< 0.001^a^
Three times the minimum wage or more	12(3.7)	101(8.0)	21(13.9)	134(7.7)	
Marital Status					
Married	246(75.7)	984(77.6)	117(77.5)	1347(77.2)	0.762^a^
Single	79(24.3)	284(22.4)	34(22.5)	397(22.8)	
Employment Status					
Employed	39(12.0)	123(9.7)	19(12.6)	181(10.4)	0.311^a^
Unemployed	286(88.0)	1145(90.3)	132(87.4)	1563(89.6)	
Smoking					
Yes	69(21.2)	237(18.7)	27(17.9)	333(19.1)	0.549^a^
No	256(78.8)	1031(81.3)	124(82.1)	1409(80.9)	
Chronic Disease					
Yes	242(74.5)	912(72.0)	94(62.2)	1248(71.6)	0.020^a^
No	83(25.5)	356(28.0)	57(37.8)	496(28.4)	
Chronic Disease*					
1	103(42.5)	414(45.4)	36(38.3)	553(44.3)	
2-3	123(50.8)	412(45.2)	51(54.3)	586(47.0)	0.234^a^
4 or more	16(6.7)	86(9.4)	7(7.4)	109(8.7)	
Medication Use					
No	80(24.6)	270(21.3)	42(27.8)	392(22.5)	0.114^a^
Yes	245(75.4)	998(78.7)	109(72.2)	1352(77.5)	
Use of 5 or More Medications					
Yes	50(20.4)	218(26.3)	26(28.6)	294(25.2)	0.825^a^
No	195(79.6)	611(73.7)	65(71.4)	871(74.8)	
Sleep Duration (hours/day)	7.4 ± 1.6	7.5 ± 2.3	8.0 ± 1.9	7.5 ± 2.2	0.017^b^
≥ 7 hours of sleep					
Yes	166(51.1)	646(50.9)	88(58.3)	900(51.6)	0.229^a^
No	159(48.9)	622(49.1)	63(41.7)	844(48.4)	
Chewing Difficulty					
Yes	43(13.2)	109(8.6)	18(11.9)	170(9.7)	0.027^a^
No	282(86.8)	1159(91.4)	133(88.1)	1574(90.3)
Tooth Loss					
Yes	268(82.5)	929(73.3)	88(58.3)	1285(73.7)	< 0.001^a^
No	57(17.5)	339(26.7)	63(41.7)	459(26.3)	
Age (years) (X̄ ± SD)	70.5 ± 5.4	71.0 ± 5.3	71.1 ± 6.0	70.9 ± 5.5	0.369^b^ 0.007^b^
Height (cm)	162.7 ± 9.5	164.0 ± 9.6	165.7 ± 8.1	164.6 ± 7.3	
Body Weight (kg)	76.5 ± 14.3	78.2 ± 13.4	79.5 ± 13.5	78.0 ± 14.0	0.058^b^
Waist Circumference (cm)	100.3 ± 15.1	101.2 ± 13.0	101.1 ± 14.5	101.0 ± 13.6	0.571^b^
Hip Circumference (cm)	107.8 ± 14.3	107.5 ± 13.4	107.8 ± 13.2	107.7 ± 14.0	0.973^b^
Waist-to-Hip Ratio	0.93 ± 0.09	0.94 ± 0.08	0.94 ± 0.09	0.95 ± 0.07	0.638^b^
MUAC	31.5 ± 4.7	31.8 ± 4.4	31.5 ± 4.5	31.7 ± 7.9	0.731^b^
BMI (kg/m^2^)	29.3 ± 6.0	29.5 ± 5.4	29.5 ± 5.2	29.5 ± 5.5	0.769^b^
BMI categorized, n(%)					
Underweight	3(0.9)	5(0.4)	1(0.6)	9(0.5)	
Normal	67(20.6)	192(15.1)	27(17.8)	286(16.4)	0.136^a^
Overweight	115(35.4)	429(33.9)	52(34.4)	596(34.2)	
Obese	140(43.1)	642(50.6)	71(47.2)	853(48.9)	

*p* < 0.05 significant level;

a, Pearson Chi-square test;

b, Kruskal Wallis H test; MUAC, Mid-upper arm circumference; BMI, Body Mass Index,

* Percentages for the “Chronic Disease” subgroups (1, 2–3, 4 or more) were calculated based on the total number of participants with at least one chronic disease (*n* = 1248).

## 3 Results

A total of 1,744 individuals participated in the study, of whom 50.2% were female (*n* = 876), and the mean age of the participants was 70.9 ± 5.5 years (Table 1). There was no statistically significant difference between gender and DDS categories (*p* = 0.351). Height was significantly higher in the high DDS group compared to the low and middle DDS groups (*p* = 0.007), whereas body weight showed no significant difference among the groups (*p* = 0.058). As education level and household income increased, individuals’ DDS scores also increased significantly (*p* < 0.05). Participants in the high DDS group were found to have a lower prevalence of chronic diseases compared to those with low and moderate DDS groups (*p* < 0.05). However, there was no statistically significant difference between the number of chronic diseases and DDS. Additionally, the average daily sleep duration of participants with high DDS was 8.0 ± 1.9 h, whereas it was 7.4 ± 1.6 h in the low DDS group, and this difference was found to be statistically significant (*p* < 0.05). Participants with low DDS had a significantly higher rate of chewing problems and tooth loss compared to those with high DDS (*p* < 0.05).

A modest pattern was observed where the proportion of independent individuals increased in tandem with dietary diversity, reaching its peak at 80.1% in the high DDS group compared to 70.8% in the low DDS group. Conversely, partial dependence was notably more prevalent among participants with low dietary diversity (28.9%) than those with high diversity (18.6%). However, this difference between the categorized Katz ADL and DDS showed a statistically borderline significance (*p* = 0.042). Regarding the phytonutrient scale, both mean scores and categorical distributions showed highly significant differences across DDS groups (*p* < 0.001). Mean scores rose progressively from 24.6 ± 6.7 in the low group to 33.0 ± 8.9 in the high group. Furthermore, while only 1.8% of the low DDS group exhibited moderate phytonutrient intake, this rate increased to 20.5% in the high DDS group. In contrast, no statistically significant differences were observed across DDS groups for mean Katz ADL scores (*p* = 0.079), MNA-SF scores (*p* = 0.229), MNA-SF categories (*p* = 0.369), EFS (*p* = 0.446), frailty categories (*p* = 0.709), SMMSE scores (*p* = 0.067), or SMMSE categories (*p* = 0.527) (Table 2).

**TABLE 2:** Activities of Daily Living, Malnutrition, Frailty, Cognitive Function, and Phytonutrients Intake of Participants According to Dietary Diversity Score.

	Dietary Diversity Score				
Variables	Low n:325,18.6%	Moderate n:1268, 72.7%	High n:151, 8.6%	Total n:1744	p
Katz ADL (X̄ ± SD)	5.60 ± 0.6	5.68 ± 0.7	5.75 ± 0.7	5.68 ± 0.7	0.079^b^
Katz ADL categorized, n(%)					
Dependent	1(0.3)	14(1.1)	2(1.3)	17(1.0)	
Partially dependent	94(28.9)	286 (22.6)	28(18.6)	408(23.4)	**0.042^c^**
Independent	230(70.8)	968(76.3)	121(80.1)	1319(75.6)	
MNA-SF score (X̄ ± SD)	12.3 ± 1.8	12.5 ± 1.7	12.7 ± 1.7	12.5 ± 1.7	0.229^b^
MNA-SF score. categorized, n(%)					
Malnourished	4(1.2)	24(1.9)	4(2.6)	32(1.8)	
At risk	79(24.3)	255(20.1)	27(17.9)	361(20.7)	0.369^a^
Normal	242(74.5)	989(78.0)	120(79.5)	1351(77.5)	
Edmonton Frailty scale (X̄ ± SD)	4.18 ± 2.6	4.12 ± 2.9	3.97 ± 2.9	4.12 ± 2.8	0.446^b^
Edmonton Frailty scale categorized n(%)					
Non-frail	191(58.8)	769(60.6)	93(61.6)	1053(60.4)	
Apparently vulnerable	74(22.8)	236(18.6)	27(17.9)	337(19.3)	
Mild frailty	41(12.6)	158(12.5)	19(12.6)	218(12.5)	0.709^a^
Moderately frailty	13(4.0)	59(4.7)	7(4.6)	79(4.5)	
Severely frailty	6(1.8)	46(3.6)	5(3.3)	57(3.3)	
SMMSE scale (X̄ ± SD)	21.9 ± 4.6	22.6 ± 4.5	22.7 ± 4.4	22.6 ± 4.5	0.067^b^
SMMSE scale categorized, n(%)					
None	142(43.7)	553(43.6)	74(49.0)	769(44.1)	
Mild	114(35.1)	430(33.9)	52(34.4)	596(34.2)	
Moderately	64(19.7)	275(21.7)	24(15.9)	363(20.8)	0.527^a^
Severely	5(1.5)	10(0.8)	1(0.7)	16(0.9)	
Phytonutrient scale (X̄ ± SD)	24.6 ± 6.7	29.0 ± 7.8	33.0 ± 8.9	28.5 ± 18.0	**< 0.001^b^**
Phytonutrient scale categorized, n(%)					
Negligible	64(19.7)	98(7.7)	5(3.3)	167(9.5)	**< 0.001^a^**
Low	255(78.5)	1070(84.4)	115(76.2)	1440(82.6)	
Moderate-High	6(1.8)	100(7.9)	31(20.5)	137(7.9)	

*p* < 0.05 significant level;

a, Pearson Chi-square test;

b, Kruskal Wallis H test;

c, Fisher Freeman Halton test; DDS, Dietary Diversity Score; Katz, Katz Index of Independence in Activities of Daily Living; MNA-SF, Mini Nutritional Assessment Short Form; SMMSE, Standardized Mini Mental State Examination

In our initial analyses according to DDS groups, participants in the high DDS group had significantly higher energy and nutrient intakes compared to those in the low DDS group. To eliminate the confounding effect of significantly higher energy intake observed in the high DDS group, all nutrients were adjusted for energy (nutrient intake/total energy × 1,000 kcal) (*p* < 0.001) (Table 3). Following energy adjustment, participants in the high DDS group were observed to have higher intakes of fat (g), saturated fat, cholesterol, and iron (*p* < 0.05). In contrast, participants in the low DDS group were found to have significantly higher intakes of carbohydrates (g), folate, sodium, and calcium compared to the high DDS group (*p* < 0.05).

**TABLE 3:** Daily energy, macro- and micronutrient intakes of participants according to dietary diversity score.

	Dietary Diversity Score				
Variables	Low	Moderate	High	Total	p^b^
Energy (Kcal)	1130.2 ± 389.3	1459.8 ± 478.2	1869.9 ± 580.9	1433.9 ± 507.5	**< 0.001**
CHO (g)	120.2 ± 24.4	116.4 ± 20.2	114.7 ± 20.0	117.0 ± 21.1	**0.009**
CHO (E%)	49.1 ± 9.9	47.6 ± 8.1	46.6 ± 8.1	47.8 ± 8.5	0.007
Protein (g)	38.6 ± 9.0	39.1 ± 7.8	39.4 ± 7.6	39.0 ± 8.0	0.101
Protein (E%)	15.7 ± 3.7	15.9 ± 3.1	16.2 ± 3.0	15.9 ± 3.2	0.064
Fat (g)	39.4 ± 10.6	40.8 ± 8.6	41.2 ± 7.7	40.6 ± 9.0	**0.026**
Fat (E%)	35.0 ± 9.4	36.2 ± 7.5	36.7 ± 6.9	36.0 ± 7.8	0.024
Saturated Fatty Acids (g)	6.8 ± 3.2	6.9 ± 2.6	7.2 ± 2.5	6.9 ± 2.7	**0.028**
Cholesterol (mg)	181.8 ± 137.2	215.3 ± 111.8	191.7 ± 92.4	207.0 ± 116.2	**< 0.001**
Fiber (g)	13.1 ± 4.7	13.1 ± 4.6	12.9 ± 3.7	13.1 ± 4.5	0.981
Vitamin A (μg)	712.8 ± 487.4	777.8 ± 734.4	660.8 ± 355.9	755.5 ± 669.7	0.060
Vitamin E (μg)	8.3 ± 4.2	7.9 ± 3.2	7.7 ± 2.7	7.9 ± 3.4	0.851
Vitamin B_1_ (mg)	0.57 ± 0.15	0.58 ± 0.15	0.57 ± 0.12	0.58 ± 0.15	0.628
Vitamin B_2_ (mg)	0.92 ± 0.28	0.91 ± 0.26	0.89 ± 0.24	0.91 ± 0.26	0.527
Vitamin B_6_ (mg)	0.78 ± 0.24	0.78 ± 0.22	0.79 ± 0.16	0.78 ± 0.22	0.168
Vitamin B_12_ (μg)	2.57 ± 2.55	2.67 ± 2.79	2.70 ± 2.39	2.65 ± 2.71	0.368
Folate (μg)	235.9 ± 82.2	212.3 ± 67.7	194.3 ± 47.3	215.4 ± 70.2	**< 0.001**
Vitamin C (mg)	84.2 ± 61.7	72.4 ± 43.1	71.6 ± 38.0	74.5 ± 46.9	0.258
Sodium (mg)	2096.3 ± 644.6	2028.9 ± 582.2	1946.9 ± 509.1	2034.4 ± 589.3	**0.012**
Potassium (mg)	1562.7 ± 491.8	1584.1 ± 535.5	1589.7 ± 305.9	1580.6 ± 511.4	0.119
Calcium (mg)	495.1 ± 186.3	442.0 ± 132.6	455.0 ± 133.8	453.0 ± 145.6	**< 0.001**
Magnesium (mg)	162.5 ± 58.1	163.7 ± 59.1	163.8 ± 35.6	163.5 ± 57.2	0.075
Phosphorus (mg)	661.9 ± 161.4	660.5 ± 140.7	661.7 ± 125.4	660.9 ± 143.5	0.492
Iron (mg)	5.9 ± 1.7	6.2 ± 1.7	6.2 ± 1.3	6.1 ± 1.7	**0.003**
Zinc (mg)	6.1 ± 1.7	6.0 ± 1.6	5.8 ± 1.2	6.0 ± 1.6	0.458

*p* < 0.05 significant level;

b, Kruskal Wallis H test; CHO, Carbohydrate

The correlation analysis revealed that the DDS had a significant positive association with energy intake (*r* = 0.453, *p* < 0.01), protein (g) (*r* = 0.434, *p* < 0.01), fat(g) (*r* = 0.438, *p* < 0.01), carbohydrate (g) (*r* = 0.341, *p* < 0.01), and phytonutrients intake (*r* = 0.296, *p* < 0.01). DDS showed a modest but significant positive correlation with nutritional status (MNA-SF: *r* = 0.088, *p* < 0.01) and cognitive function (SMMSE: *r* = 0.075, *p* < 0.01), while being inversely related to frailty levels (EFS: *r* = −0.060, *p* < 0.05). Notably, the EFS showed strong negative correlations with the MNA-SF score (*r* = −0.486, *p* < 0.01), SMMSE (*r* = −0.419, *p* < 0.01), and Katz ADL score (*r* = −0.434, *p* < 0.01) (Table 4).

**TABLE 4:** Spearman’s Rho correlation coefficients between dietary diversity score (DDS), geriatric assessment scales, and dietary intake parameters.

Variables	1	2	3	4	5	6	7	8	9	10	11	12	13
DDS score^1^	1	1	1	1	1	1	1	1	1	1	1	1	1
Edmonton frailty scale^2^	−0.060*												
Katz ADL Score^3^	0.028	−0.434**											
MNA-SF score^4^	0.088**	−0.486**	0.252**										
Phytonutrient scale^5^	0.296**	−0.202**	0.160**	0.209**									
SMMSE^6^	0.075**	−0.419**	0.225**	0.287**	0.126**								
Energy (kcal)^7^	0.453**	−0.135**	0.070**	0.199**	0.224**	0.084**							
Protein (g)^8^	0.434**	−0.117**	0.063**	0.144**	0.143**	0.065**	0.838**						
Protein (%)^9^	0.037	0.009	−0.016	−0.076**	−0.098**	−0.024	−0.148**	0.365**					
Fat (g)^10^	0.438**	−0.136**	0.046	0.157**	0.152**	0.089**	0.843**	0.720**	−0.114**				
Fat (%)^11^	0.119**	−0.047*	−0.036	−0.01	−0.047*	0.063**	0.042	0.047*	0.013	0.530**			
CHO (g)^12^	0.341**	−0.101**	0.079**	0.197**	0.246**	0.052*	0.877**	0.649**	−0.288**	0.523**	−0.363**		
CHO (%)^13^	−0.121**	0.045	0.034	0.043	0.081**	−0.062**	0.011	−0.174**	−0.355**	−0.446**	−0.907**	0.449**	

Bold values indicate statistically significant correlations.

*p < 0.05,

**p < 0.01.

Multinomial logistic regression models were used to identify the independent predictors of functional independence, frailty status, phytonutrients intake, MNA-SF and SMMSE. Based on preliminary analyses where significant differences between DDS groups were identified, education, household income, chronic disease, and tooth loss were included as covariates in all models (Table 5). Due to the violation of the proportional odds assumption in initial ordinal models (*p* < 0.05), multinomial regression was preferred to provide discrete odds ratios (OR) for each categorical transition.

**TABLE 5:** Multinomial logistic regression models for factors associated with Katz ADL, EFS, Phytonutrient, MNA-SF and SMMSE levels.

Variables	Model 1 Katz ADL (Ref:Independent)	Model 2 EFS (Ref:Non-Frail)	Model 3 Phytonutrient (Ref: Negligible)	Model 4 MNA-SF (Ref: Normal)	Model 5 SMMSE (Ref: None)
	Dependent Partially Dependent	Vulnerable Low Moderate High	Low Moderate-High	Malnourished At risk	Severely Moderately Mild
	OR(95%CI)	OR(95%CI)	OR(95%CI)	OR(95%CI)	OR(95%CI)
Education	0.13(0.05-0.35)* 0.66(0.56-0.79)*	0.60(0.49-0.74)* 0.35(0.27-0.46)* 0.31(0.21-0.47)* 0.11(0.06-0.19)*	1.42(1.10-1.84)* 1.37(0.97-1.92)	0.28(0.15-0.53)* 0.87(0.73-1.04)*	0.20(0.08-0.51)* 0.19(0.15-0.24)* 0.44(0.36-0.52)*
Family income	1.78(1.08-2.93)* 1.09(0.96-1.24)	0.81(0.71-0.94)* 0.90(0.76-1.06) 0.83(0.64-1.09) 0.81(0.58-1.12)	0.77(0.65-0.93)* 1.13(0.88-1.45)	0.95(0.64-1.42) 0.92(0.81-1.05)	0.31(0.13-0.74)* 0.73(0.63-0.85)* 0.76(0.67-0.86)*
Chronic disease	2.40(0.54-10.74) 1.09(0.84-1.41)	2.78(2.01-3.84)* 2.42(1.65-3.54)* 2.34(1.28-4.26)* 2.53(1.20-5.33)*	1.66(1.17-2.36)* 2.44(1.44-4.15)*	1.55(0.63-3.83) 1.36(1.03-1.79)*	1.83(0.51-6.58) 1.43(1.04-1.95)* 1.38(1.08-1.78)*
Tooth Loss	2.46(0.55-11.09) 2.18(1.61-2.95)*	1.44(1.06-1.96)* 1.48(1.02-2.16)* 1.96(1.03-3.74)* 2.25(0.98-5.17)	0.74(0.49-1.12) 0.94(0.54-1.66)	1.05(0.44-2.50) 1.24(0.93-1.65)	3.57(0.46-27.94) 0.91(0.66-1.26) 0.95(0.73-1.24)
DDS-Low	0.24(0.20-2.85) 1.40(0.86-2.29)	0.88(0.52-1.50) 0.67(0.35-1.25) 0.52(0.20-1.40) 0.30(0.09-1.09)	0.17(0.07-0.43)* 0.02(0.00-0.05)*	0.39(0.09-1.65) 1.23(0.75-2.02)	1.15(0.13-10.41) 0.91(0.50-1.66) 0.81(0.51-1.28)
DDS-Moderate	0.79(0.17-3.71) 1.11(0.72-1.74)	0.82(0.51-1.32) 0.77(0.44-1.33) 0.73(0.32-1.69) 0.73(0.27-1.99)	0.46(0.18-1.16) 0.16(0.06-0.43)*	0.63(0.21-1.88) 1.04(0.67-1.62)	0.85(0.10-6.97) 1.28(0.75-2.17) 0.96(0.64-1.43)
*p* value	**< 0.001**	**<0.001**	**< 0.001**	**<0.001**	**< 0.001**
χ^2^	95.68	312.12	124.16	40.42	365.65
Nagelkerke R^2^	0.077	0.183	0.100	0.033	0.213

Reference categories for outcomes: Independent (Katz ADL), Non-Frail (Edmonton frailty scale), and Negligible (Phytonutrient). Reference category for DDS group is DDS-High.

*p < 0.05 significant level

In Model 1, higher education level was a significant protective factor against functional dependence; participants with higher education were 87% less likely to be categorized as dependent (OR = 0.13, *p* < 0.05). Tooth loss was significantly associated with partial dependence, with affected individuals having 2.18-fold higher odds of being classified in this category (*p* < 0.05). Additionally, higher family income was associated with increased odds of being in the dependent category (OR = 1.78, *p* < 0.05).

In Model 2, chronic disease and tooth loss were significant factors associated with frailty. Chronic disease increased the odds of vulnerability and frailty across all categories, with the highest risk observed in the vulnerable group (OR = 2.78, *p* < 0.05). Similarly, tooth loss was associated with higher odds of vulnerability (OR = 1.44), mild frailty (OR = 1.48), and moderate frailty (OR = 1.96) (*p* < 0.05). While lower DDS showed a trend toward increased frailty, it did not reach statistical significance in this specific model after adjusting for all covariates.

In Model 3, a significant association was observed between DDS and phytonutrient intake. Participants in the low DDS group were 83% less likely to achieve low phytonutrient intake (OR = 0.17) and 99% less likely to reach high levels (OR = 0.01) compared to the high DDS group (*p* < 0.001). Furthermore, the presence of chronic disease was associated with higher odds of both low (OR = 1.66) and moderate-high phytonutrient levels (OR = 2.44) (*p* < 0.05). Higher education level (OR = 1.42) and lower family income (OR = 0.77) were also significantly associated with low phytonutrient levels (*p* < 0.05).

In Model 4, nutritional status was primarily influenced by education and chronic disease. Higher education emerged as a significant protective factor against malnutrition; participants with higher education were 72% less likely to be categorized as malnourished (OR = 0.28, *p* < 0.05). The presence of chronic disease significantly increased the likelihood of being “at risk” of malnutrition by 1.36-fold (*p* < 0.05). Other factors, including DDS and family income, did not reach statistical significance in predicting nutritional risk after adjusting for these key covariates.

In Model 5, SMMSE showed the strongest associations with socioeconomic covariates. Education was the most robust protective factor against cognitive impairment; individuals with higher education levels were 80% less likely to have severe cognitive decline (OR = 0.20, *p* < 0.05) and 81% less likely to have moderate decline (OR = 0.19, *p* < 0.05). Family income also served as a significant protective covariate, with higher income levels associated with a reduced likelihood of severe (OR = 0.31) and moderate (OR = 0.73) cognitive impairment. Chronic disease was associated with a 1.43-fold increased risk of moderate cognitive impairment (*p* < 0.05). After adjusting for all covariates, no independent effect of DDS on SMMSE was found.

## 4 Discussion

This study aimed to evaluate the relationships between DDS and frailty, cognitive function, activities of daily living, phytonutrient intake, and nutritional status among community-dwelling Turkish individuals aged 65 years and over. The study involved a total of 1,744 participants, of whom 50.2% were female. Participants with high DDS scores were found to have higher education and income levels, and a lower prevalence of chronic diseases. Additionally, participants in the high DDS group were found to have longer sleep durations and fewer tooth loss and chewing difficulty. DDS was positively correlated with energy intake, protein (g), fat (g), carbohydrate (g), and phytonutrient intake, and showed weak but significant positive correlations with MNA-SF and SMMSE, while it showed a negative correlation with frailty. According to multinomial logistic regression analysis, no significant association was found between DDS and activities of daily living, frailty, MNA-SF, and SMMSE. However, participants with lower DDS values were less likely to have higher phytonutrient intake.

Dietary diversity plays a critical role in overall health and aging-related health outcomes in older populations and is inversely associated with all-cause mortality rates (33). A study conducted in Thailand reported that the average DDS increased with higher education and income levels and decreased with advancing age (34). In China, among adults aged 65 years and older, male participants were found to have a higher likelihood of better dietary diversity compared to females (35). A study conducted in West Bengal found that women, participants aged 80 and over, and individuals with low education levels and income had lower DDS values (36). A study involving 5,260 older adults living in China reported that 47.2% of participants aged 60–69 had low dietary diversity, whereas this rate increased to 53.1% among individuals aged 90 and above (37). However, the same study also found that women, as well as participants with low education levels and income, had lower DDS rates. In our study, we observed that lower DDS was significantly associated with lower education levels and household income (*p* < 0.001). However, contrary to some previous literature, no statistically significant differences were found in DDS distribution regarding gender (*p* = 0.351) and age groups (*p* = 0.511). The complex interplay of socioeconomic factors, age-related physiological changes, cultural norms influencing food consumption, and gender-specific health conditions may play a role in these results.

The aging process, characterized by a combination of physiological, cognitive, emotional, and behavioral changes, adversely affects nutritional status and leads to malnutrition. Cognitive decline, depression, social isolation, and socioeconomic challenges also contribute to malnutrition (38). Kong et al. reported that 67% of participants aged 60 and older had normal MNA-SF scores, 32% were at risk of malnutrition, and less than 1% were malnourished. Additionally, they observed that the group with low dietary diversity had significantly higher rates of malnutrition and risk of malnutrition compared to the other groups (39). In a study conducted among older Arab adults, 16.9% of participants were found to be malnourished or at risk of malnutrition. The proportion of individuals in this group with high or moderate DDS categories was lower compared to the well-nourished group (40). In their study conducted in China among participants aged 60 and older, Hua et al. found that 32.4% of participants were malnourished or at risk of malnutrition. They reported that 64.9% of those with moderate or insufficient dietary diversity were malnourished or at risk of malnutrition (41). In this study, 1.8% of participants were found to be malnourished, and 20.7% were at risk of malnutrition. The difference in mean MNA-SF scores between DDS groups was not statistically significant (*p* > 0.05). Although correlation analysis revealed a significant relationship between DDS and MNA-SF (*r* = 0.088, *p* < 0.01), this relationship was highly inconsistent and did not persist in the model adjusted for confounding factors. DDS reflects the diversity of food groups consumed and does not assess actual dietary intake in terms of quantity or adequacy. In contrast, the MNA-SF is a multidimensional nutritional assessment tool that includes appetite, recent weight loss, eating habits, mobility, and psychological status. Additionally, socioeconomic and clinical factors may influence nutritional status in older adults. Considering the complexity of variables affecting nutritional status in older adults; these factors are thought to play a role in the lack of a significant relationship between DDS and MNA-SF in our study.

Chronic diseases and multi-morbidity are common problems in older adults and lead to impairments in physiological functioning and increase the risk of frailty. Nutrition is a modifiable factor in frailty, which plays a critical role in the emergence, development, and reversal of frailty in older adults (42). Wang et al. observed a 26% reduction in frailty risk among participants with the highest DDS compared to those with the lowest DDS. Additionally, each one-unit increase in DDS was associated with a 5% decrease in frailty risk (11). In a study conducted among older Japanese adults, the mean DDS was reported as 3.9 ± 2.1 in frail individuals, 4.3 ± 2.2 in pre-frail individuals, and 4.5 ± 2.2 in robust older adults (43). The combination of frailty and low DDS contributes to adverse health outcomes. Older adults with lower DDS combined with pre-frailty or frailty were reported to have a 2.15 times higher risk of cognitive impairment compared to those without frailty or with higher DDS (15). Otsuka et al. found a 0.79-point decrease in SMMSE score for each 1 standard deviation (SD) increase in dietary diversity score (44). Zhong et al. investigated the effect of DDS on cognitive frailty (CF) and found that higher DDS was associated with lower CF, while a decrease in DDS over time further increased the risk of CF (45). In our study, lower DDS was associated with higher frailty scores in bivariate analyses; however, this association lost statistical significance after adjustment for potential confounders in the multivariable model. The selection of participants using convenience sampling, their relatively similar socioeconomic and sociocultural characteristics, and the exclusion of individuals with severe neurocognitive impairment may explain this finding regarding the relationship between frailty and DDS. The difference between DDS groups in SMMSE scale and SMMSE scale categorization was not statistically significant. Additionally, although a relationship was found between DDS and SMMSE in the correlation analysis (*r* = 0.075, *p* < 0.01), this inconsistent relationship lost its statistical significance when evaluated with multiple logistic regression analysis adjusted for confounding factors. This finding suggests that DDS may not be independently associated with cognitive function. The variability in findings across studies may be explained by differences in participants’ age distribution, socioeconomic status, dietary habits, levels of functional dependency, as well as the assessment methods used to evaluate cognitive function and dietary diversity.

With aging, losses in activities of daily living (ADL), functional capacity, and abilities may gradually lead to dependency (18). However, evidence regarding the association between DDS and ADL remains limited. A study involving older adults individuals in Taiwan showed that as DDS increased, the likelihood of experiencing difficulty in various mobility activities decreased, and 6the risk of ADL decline was also reduced by 40% (46). In their 40-month longitudinal study, Kiuchi et al. reported disability rates of 23.3 and 16.9% among participants (aged ≥ 65 years) with low and high DDS, respectively. They also found that high dietary diversity at baseline was associated with a lower risk of incident disability (47). Similarly, in Zhang et al.’s cohort study, participants with low DDS scores exhibited higher rates of disability, and an inverse relationship between DDS and the risk of ADL disability was reported (18). However, in a long-term cohort study, participants with low dietary diversity had an odds ratio of 1.283 for instrumental activities of daily living (IADL) limitations; whereas, after model adjustments, dietary diversity was reported to have no significant effect on the development of IADL limitations (48). In this study, the proportion of independent individuals tended to be lower among participants with low DDS scores compared to those with high DDS scores, whereas the proportion of partially dependent individuals appeared lower in the high DDS group. However, these differences, based on the categorized ADL, indicate only a borderline significant (*p* = 0.042). Furthermore, no significant association was observed between DDS and ADL in the correlation analysis and multiple logistic regression model. Several factors, including the type of ADL scale used, participants’ age, and sociodemographic characteristics, may play a role in the variations in dependency rates reported across studies.

Aging is associated with a range of health conditions, including dysphagia, depression, dementia, and decline in motor function, which can adversely affect nutritional status. Consequently, the deterioration in nutritional status and reduced dietary diversity may place the majority of older individuals at risk of inadequate and unbalanced nutrition (12, 18, 49). Zhang et al. reported that intake of protein, fat, dietary fiber, vitamin A, riboflavin, niacin, vitamin C, vitamin E, calcium, phosphorus, potassium, magnesium, iron, zinc, and selenium was positively correlated with DDS; conversely, higher DDS was inversely correlated with carbohydrate, sodium, and manganese intake (18). In Japanese adults with low DDS, energy, protein, fat, and fiber intakes were found to be lower compared to those with high DDS, while carbohydrate intake was similar across groups (12). Otsuka et al. reported that older adults in Japan with low DDS had lower intakes of energy, macro-, and micronutrients (50). In our study, it was determined that participants with high DDS scores had higher energy intakes. After adjusting for energy intake, participants with high DDS scores had higher intakes of fat (g), saturated fat, cholesterol, and iron, and lower intakes of carbohydrates (E%), folate, calcium, and sodium compared to participants with low DDS scores (*p* < 0.05). Furthermore, we identified significant positive correlations between DDS and energy (*r* = 0.453; *p* < 0.01), protein (*r* = 0.434; *p* < 0.01), fat (*r* = 0.438; *p* < 0.01), and carbohydrate (g) (*r* = 0.341; *p* < 0.01) intakes. In our study, unlike literature, those with a high DDS score had higher intakes of fat (g), saturated fat, and cholesterol. This finding may be explained by the characteristics of traditional Turkish cuisine, which often includes foods with relatively high fat content.

An increase in dietary diversity is considered to enhance phytonutrient intake (16). Overall, the promotion of a diverse dietary pattern rich in phytonutrients is known to support healthy aging (51). However, studies investigating the relationship between dietary diversity and phytonutrient intake specifically in the older adults population are lacking. In our study, a significant positive correlation was identified between the DDS and phytonutrient intake. In parallel, multivariate analysis revealed that DDS remained significantly associated with phytonutrient scores; participants with lower DDS levels were less likely to have high phytochemical intake.

One of the strengths of this study is that the questionnaire items were easy for older adults to understand and respond to. Another strength is that dietary records were accurately collected with the assistance of a trained dietitian. However, the study has some limitations. Dietary intake data are based on participants’ self-reported two-day dietary recall, which may lead to underreporting of nutrient intake due to recall bias and reporting errors. This method was preferred due to its low research burden, time efficiency, and feasibility in older populations. However, this is a commonly used and considered valid approach to assess nutritional intake in population-based studies. Additionally, limitations include cross-sectional design, seasonal data collection, and potential confounding due to the high prevalence of obesity. Furthermore, the analysis did not account for household structure, health literacy, or urban-rural distinctions. Finally, since participation in the study was voluntary, it is unknown whether the sample fully represents the characteristics of the general population.

## 5 Conclusion

In conclusion, in this study, no significant association was found between DDS and nutritional status, activities of daily living, functional and cognitive frailty, whereas a significant association was observed between DDS and phytonutrient intake. It is considered that regular monitoring of nutritional status and interventions aimed at increasing dietary diversity in the older adults population may support healthy aging.

## Data Availability

The datasets presented in this article are not readily available due to restrictions (e.g., their containing information that could compromise the privacy of research participants). Requests to access the datasets should be directed to volkan.ozkaya@ksbu.edu.tr.
